# Genome-Wide Screen for Haploinsufficient Cell Size Genes in the Opportunistic Yeast *Candida albicans*

**DOI:** 10.1534/g3.116.037986

**Published:** 2016-12-28

**Authors:** Julien Chaillot, Michael A. Cook, Jacques Corbeil, Adnane Sellam

**Affiliations:** * Infectious Diseases Research Centre, Centre Hospitalier Universitaire (CHU) de Québec Research Center, Université Laval, Quebec City, Quebec, Canada; ‡ Department of Molecular Medicine, Université Laval, Quebec City, Quebec, Canada; § Department of Microbiology, Infectious Disease and Immunology, Faculty of Medicine, Université Laval, Quebec City, Quebec, G1V 4G2 Canada; † Centre for Systems Biology, Samuel Lunenfeld Research Institute, Mount Sinai Hospital, Toronto, G1V 4G2 Canada

**Keywords:** *Candida albicans*, cell size, haploinsufficiency, Start control

## Abstract

One of the most critical but still poorly understood aspects of eukaryotic cell proliferation is the basis for commitment to cell division in late G1 phase, called Start in yeast and the Restriction Point in metazoans. In all species, a critical cell size threshold coordinates cell growth with cell division and thereby establishes a homeostatic cell size. While a comprehensive survey of cell size genetic determinism has been performed in the saprophytic yeasts *Saccharomyces cerevisiae* and *Schizosaccharomyces pombe*, very little is known in pathogenic fungi. As a number of critical Start regulators are haploinsufficient for cell size, we applied a quantitative analysis of the size phenome, using elutriation-barcode sequencing methodology, to 5639 barcoded heterozygous deletion strains of the opportunistic yeast *Candida albicans*. Our screen identified conserved known regulators and biological processes required to maintain size homeostasis in the opportunistic yeast *C. albicans*. We also identified novel *C. albicans*-specific size genes and provided a conceptual framework for future mechanistic studies. Interestingly, some of the size genes identified were required for fungal pathogenicity suggesting that cell size homeostasis may be elemental to *C. albicans* fitness or virulence inside the host.

In eukaryotic species, growth and division are coupled at Start (Restriction Point in metazoans), the point in late G1 at which the cell commits to the next round of division (Jorgensen and Tyers 2004). Cells must grow to reach a critical size threshold at Start and thereby establish a homeostatic cell size. Pioneering studies in the eukaryotic model *Saccharomyces cerevisiae* revealed that a large proportion of the genome (>10%) and of cellular functions impact on the size of cells, in processes ranging from ribosome biogenesis (*Ribi*) and mitochondrial function, to signal transduction and cell cycle control (Jorgensen *et al.* 2002; Zhang *et al.* 2002; Soifer and Barkai 2014). While follow-on studies revealed many crucial players in size regulation, such as the G1 repressor Whi5 and the *Ribi* master regulators Sch9 and Sfp1, both the central mechanism by which cells sense their size and the means by which they alter their size set-point to meet environmental demands remain elusive (Turner *et al.* 2012).

*Candida albicans* is a diploid ascomycete yeast that is an important commensal and opportunistic pathogen in humans colonizing primarily mucosal surfaces, gastrointestinal and genitourinary tracts, and skin (Berman and Sudbery 2002). Interest in *C. albicans* is not limited to understanding its function as a pathogenic organism, as it has an ecological niche that is obviously distinct from the classic model ascomycete *S. cerevisiae*. *C. albicans* has served as an important evolutionary milepost with which to assess conservation of biological mechanisms. Recent investigations uncovered an extensive degree of rewiring of fundamental signaling and transcriptional regulatory networks as compared to *S. cerevisiae* and other fungi (Lavoie *et al.* 2009; Sellam *et al.* 2009; Blankenship *et al.* 2010; Homann *et al.* 2009; Sandai *et al.* 2012).

Haploinsufficiency is a phenotypic feature wherein a deletion of one allele in a diploid genome leads to a discernable phenotype. In eukaryotes, a number of critical size regulators, such as the G1 cyclin Cln3 and the AGC kinase Sch9 in *S. cerevisiae*, and the Myc oncogene in *Drosophila melanogaster* are haploinsufficient (Jorgensen *et al.* 2002; Barna *et al.* 2008; Sudbery *et al.* 1980). Here, we exploited gene haploinsufficiency to identify genes and biological process that influence size control in *C. albicans*. Given the importance of *C. albicans* as an emerging eukaryotic model, very little is known regarding the genetic networks that control size homeostasis in this opportunistic yeast. A systematic screen using elutriation-based size fractioning (Cook *et al.* 2008) coupled to barcode sequencing (Bar-seq) identified 685 genes (10% of the genome) that influenced size control under optimal growth conditions. While *C. albicans* and *S. cerevisiae* share the morphological trait of budding, and core cell cycle and growth regulatory mechanisms (Berman 2006; Cote *et al.* 2009), a limited overlap was obtained when comparing the size phenome of both yeasts. This genome-wide survey will serve as a primary entry point into the global cellular network that couples cell growth and division in *C. albicans*.

## Materials and Methods

### Strains and growth conditions

*C. albicans* SC5314 and CAI4 (*ura3*::imm434/*ura3*::imm434 *iro1/iro1*::imm434) (Fonzi and Irwin 1993) wild-type (WT) strains, and mutants of the Merck DBC (Double BarCoded) heterozygous diploid collection (Xu *et al.* 2007) were routinely maintained at 30° on YPD (1% yeast extract, 2% peptone, 2% dextrose, and 50 mg/ml uridine) or synthetic complete (0.67% yeast nitrogen base with ammonium sulfate, 2.0% glucose, and 0.079% complete supplement mixture) media. The Merck DBC collection is available for public distribution through the NRC’s Royalmount Avenue Research Facility (Montreal, Canada).

### Combination of C. albicans mutants into a single pool

A sterilized 384-well pin tool was used to transfer DBC mutant cells into Nunc Omni Trays containing YPD-agar, and colonies were grown for 48 hr at 30°. Missing or slow growing colonies were grown separately by repinning 3.5 µl from the initial liquid cultures. Each plate was overlaid with 5 ml of YPD, and cells were resuspended using Lazy-L spreader and harvested by centrifugation for 5 min at 1800 × *g*. The obtained cell pellet was resuspended in 20 ml fresh YPD and DMSO was added to 7% (v/v). Mutant pools were aliquoted and stored at −80°.

### Cell size selection by centrifugal elutriation

The mutant pool was size-fractioned using centrifugal elutriation with the Beckman JE-5.0 elutriation system. This technique separates cells on the basis of size. A tube of pooled mutant population was thawed on ice and used to inoculate 2 l of YPD at an OD_595_ of 0.05. Mutant cells were grown for four generations at 30° under agitation to reach ∼5 × 10^10^ cells. Cells were then pelleted by centrifugation and resuspended in 50 ml fresh YPD. To disrupt potential cell clumps and separate weakly attached mother and daughter cells, the 50 ml pooled cells were gently sonicated twice for 30 sec. The resuspended cells were directly loaded into the elutriator chamber of the Beckman JE-5.0 elutriation rotor. A 1 ml sample of cells was retained separately as a pre-elutriated cell fraction. The flow rate of the pump was set to 8 ml/min to ensure the loading of cells. To elute small cell size mutant fractions, the pump flow rate was increased in a step-wise fashion (in 2–4 ml/min increments). For each flow rate, a volume of 250 ml was collected from the output line of the rotor.

### Bar-seq

Bar-seq was performed using Illumina HiSeq2500 platform. Genomic DNA was extracted from each cell fraction using YeaStar kit (Zymo Research). The 20-bp UpTag barcode of each strain were amplified by PCR (Xu *et al.* 2007). Primers used for PCR recognize the common region of each barcode and contain the multiplexing tag and sequences required for hybridization to the Illumina flow cell. PCR products were purified from an agarose gel using the QIAquick Gel Extraction kit (Qiagen) and quantified by QuantiFluor dsDNA System (Promega). Bar-seq data were processed as following: after filtering out low frequency barcode counts, the complete set of replicate barcode reads were normalized using a cyclic loess algorithm (R package “limma”). Reads from individual elutriation fractions, relative to the pre-elutriation population, were further M-A loess normalized and converted to *Z* scores.

### Confirmation of cell size phenotypes

Cell size determination was performed using a Z2-Coulter Counter channelizer (Beckman Coulter). The Coulter principal is based on electrical impedance measurement, which is proportional to cell volume (Coulter 1953). *C. albicans* cells were grown overnight in YPD at 30°, diluted 1000-fold into fresh YPD and grown for 5 hr at 30° to reach a final density of 5 × 10^6^–10^7^ cells/ml, a range in which size distributions of the different WT strain used in this study do not change. A total of 100 µl of exponentially growing cells was diluted in 10 ml of Isoton II electrolyte solution, sonicated three times for 10 sec and the distribution measured at least three times on a Z2-Coulter Counter. Size distribution data were normalized to cell counts in each of 256 size bins and size reported as the peak median value for the distribution. Data analysis and size distribution visualization were performed using the Z2-Coulter Counter AccuComp software.

### Determination of critical cell size

Critical sizes of *cln3*/*CLN3*, *cdc28*/*CDC28* and *sch9*/*SCH9* mutants were determined using budding index as a function of size. G1 daughter cells were obtained using the JE-5.0 centrifugal elutriation system (Beckman Coulter) as described previously (Tyers *et al.* 1993). *C. albicans* G1-cells were released in fresh YPD medium and fractions were harvested at an interval of 10 min to monitor bud index. Additional fractions were collected to assess transcript levels of the *RNR1* and *ACT1* as cells progressed along the G1 phase.

### Real-time quantitative PCR

A total of 10^8^ G1 phase cells were harvested, released into fresh YPD medium,, grown for 10 min prior to harvesting by centrifugation and stored at −80°. Total RNA was extracted using the RNAeasy purification kit (Qiagen) and glass bead lysis in a Biospec Mini 24 bead-beater, as previously described (Sellam *et al.* 2009). cDNA was synthesized from 2 µg of total RNA using the SuperScript III Reverse Transcription system [50 mm Tris-HCl, 75 mm KCl, 10 mm dithiothreitol, 3 mm MgCl_2_, 400 nm oligo(dT)_15_, 1 m random octamers, 0.5 mm dNTPs, and 200 U Superscript III reverse transcriptase]. The total volume was adjusted to 20 µl, and the mixture was then incubated for 60 min at 42°. Aliquots of the resulting first-strand cDNA were used for real-time quantitative PCR (qPCR) amplification experiments. qPCR was performed using the iQ 96-well PCR system for 40 amplification cycles and QuantiTect SYBR Green PCR master mix (Qiagen). Transcript levels of *RNR1* were estimated using the comparative Ct method, as described by Guillemette *et al.* (2004), and the *C. albicans ACT1* open reading frame as a reference. The primer sequences were as follows: *RNR1*-forward: 5′-GACTATCTACCATGCTGCTGTTG-3′; *RNR1*-reverse: 5′-GGTGCAACCAACAAGGAGTT-3′; *ACT1*-forward: 5′-GAAGCCCAATCC AAAAGA-3′; and *ACT1*-reverse: 5′-CTTCTGGAGCAACTCTCAATTC-3′.

### Gene ontology analysis

Gene ontology (GO) term enrichment of size mutants was determined using the Generic GO Term Finder tool (http://go.princeton.edu/cgi-bin/GOTermFinder), with multiple hypothesis correction (Boyle *et al.* 2004). Descriptions related to gene function in Supplemental Material, Table S2 were extracted from the *Candida* Genome Database (CGD) database (Inglis *et al.* 2012). Information related to gene essentiality/dispensability was taken from O’Meara *et al.* (2015) and the CGD database.

### Data availability

The authors state that all data necessary for confirming the conclusions presented in the article are represented fully within the article.

## Results and Discussion

### The Cln3-Cdc28 kinase complex and Sch9 are haploinsufficient for cell size

**Figure 1:**
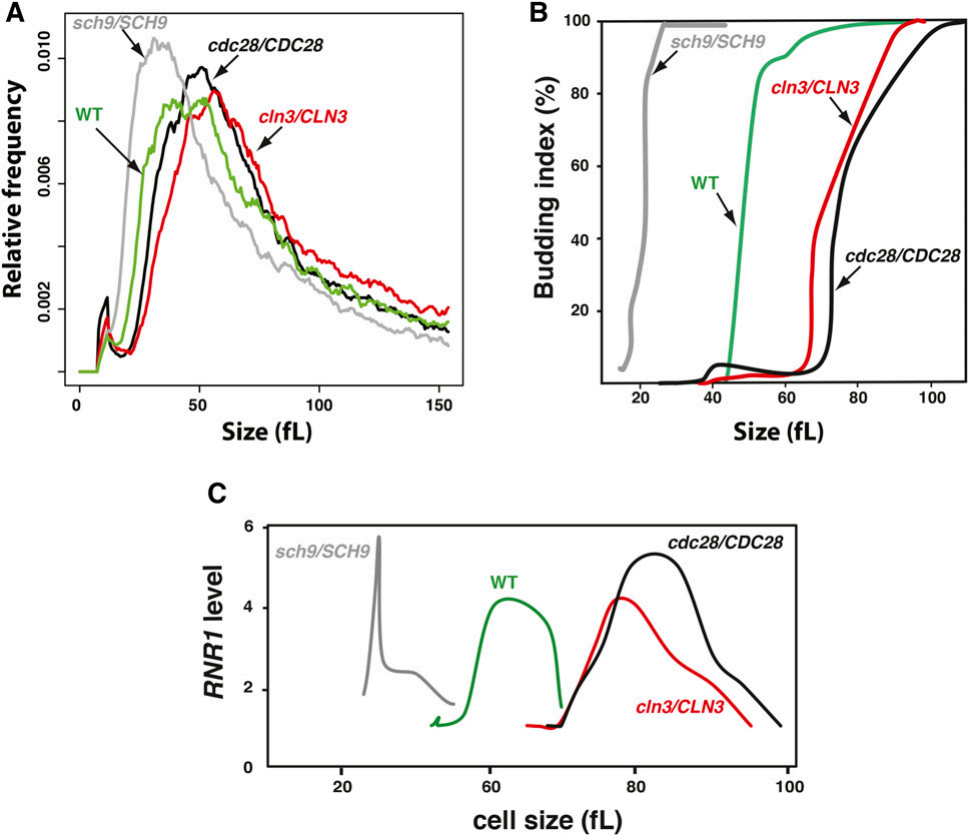
The Cln3-Cdc28 kinase complex and the AGC kinase Sch9 control Start in *C. albicans*. (A) Size distributions of the WT strain (CAI4) as compared to *lge* mutants *cln3/CLN3* and *cdc28/CDC28*, as well as the *whi* mutant *sch9/SCH9*. (B and C) Start is delayed in *cln3/CLN3* and *cdc28/CDC28* and accelerated in *sch9/SCH9*. (B) Elutriated G1 phase daughter cells were released into fresh media and monitored for bud emergence as a function of size. (C) G1/S transcription. *RNR1* transcript level was assessed by quantitative real-time PCR and normalized to *ACT1* levels.

In *S. cerevisiae*, a number of critical Start regulators are haploinsufficient for cell size, including the rate-limiting G1 cyclin Cln3 and a number of essential ribosome biogenesis factors, such as the AGC kinase Sch9 (Sudbery *et al.* 1980; Jorgensen *et al.* 2002). To test whether size haploinsufficiency exists in *C. albicans* homologs, size distributions of the heterozygous mutants of AGC kinase Sch9, the cyclin Cln3 G1, and its associated cyclin-dependent kinase Cdc28 were examined. Both *cln3/CLN3* and *cdc28/CDC28* showed an increase of size as compared to their congenic parental strain, with median sizes 13% (59 fl) and 19% (62 femtolitre) larger than the WT strain (52 fl), respectively (Figure 1A). As in *S. cerevisiae*, *sch9/SCH9* exhibited a reduced size of ∼23% (40 fl) as compared to WT.

Two hallmarks of Start, namely SBF-dependent transcription and bud emergence, were delayed in both *cln3/CLN3* and *cdc28/CDC28* and accelerated in *sch9/SCH9*, demonstrating that the Cln3-Cdc28 complex and Sch9 regulate the cell size threshold at Start. The *cln3/CLN3* mutant passed Start after growing to 92 fl, 24% higher than the parental WT cells, which budded at 74 fl (Figure 1B). Similarly, *cdc28/CDC28* reached Start at 105 fl, which is 41% higher than WT. The onset of G1/S transcription was delayed in both mutants, as judged by the expression peak of the G1-transcript *RNR1* (Figure 1C). The small mutant *sch9/SCH9* passed Start at 30 fl, a size 60% smaller than the WT, and displayed accelerated G1/S transcription (Figure 1, B and C). These data demonstrate that, as in *S. cerevisiae*, size haploinsufficiency in *C. albicans* can be used to screen for dosage-dependent regulators of growth and division at Start.

### A high-throughput screen for cell size haploinsufficiency

**Figure 2:**
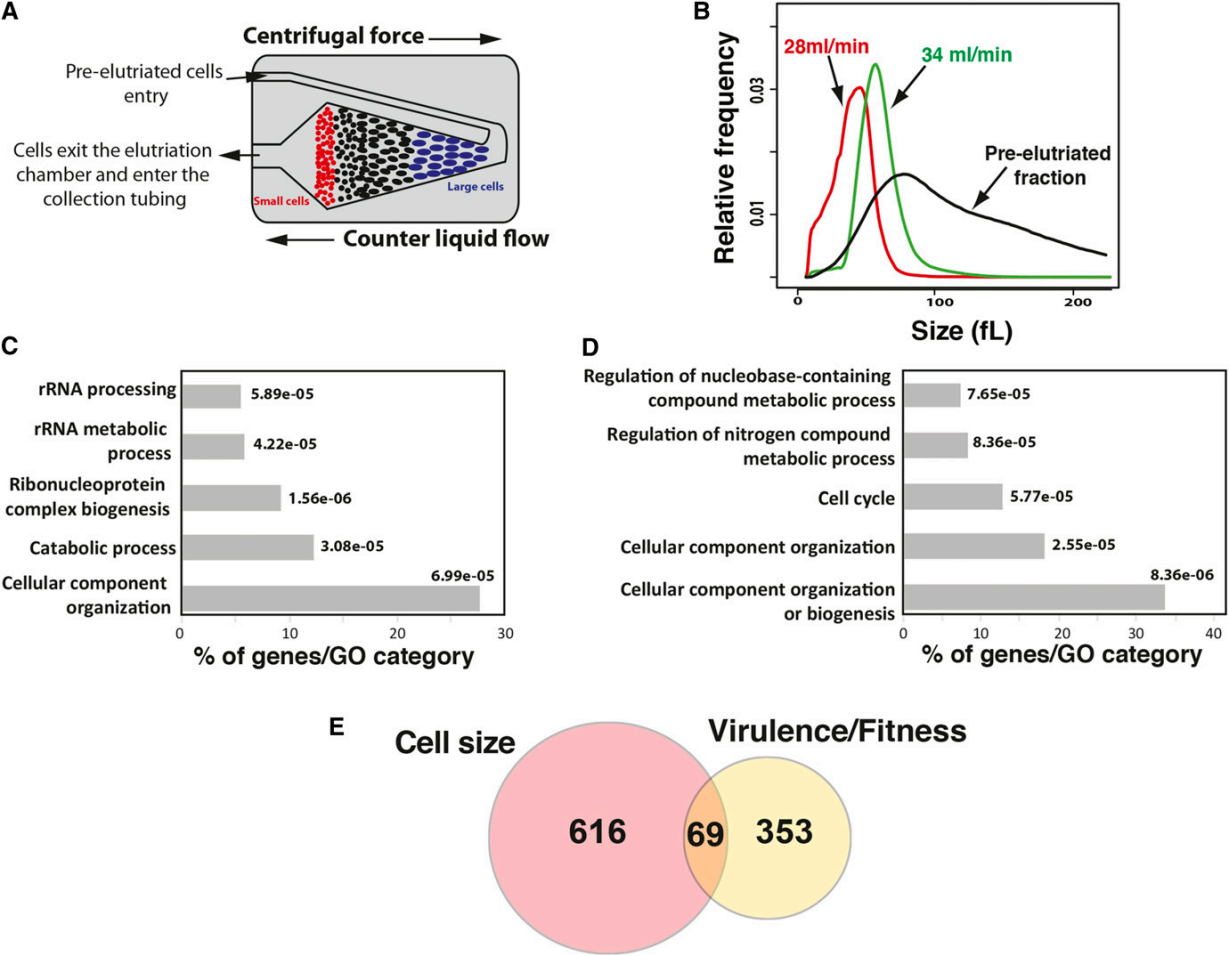
Systematic cell size screen using molecular barcode elutriation and Bar-seq. (A) Centrifugal elutriation separates cells on the basis of size. Progressive increase of the rate of flow of liquid medium counter to the direction of centrifugal force elutes yeast cells of increasingly larger size from the chamber. (B) Size distributions of pooled DBC mutants before (pre-elutriated) and after elutriation of two small cell fractions (28 and 34 ml/min). (C and D) GO terms enrichment of *whi* (C) and *lge* (D) mutants (*P* > 1e−05). GO analysis was performed using GOTermFinder (http://go.princeton.edu/cgi-bin/GOTermFinder). (E) Overlap between *C. albicans* genes haploinsufficient for cell size and those affecting virulence phenotypes. Avirulent mutant phenotypes were obtained from CGD based on decreased competitive fitness in mice and/or reduced invasion and damage to host cells.

To identify all dosage-sensitive regulators of size in *C. albicans*, a genome-wide screen was performed where pooled mutants were separated based on their size by centrifugal elutriation and their abundance determined by Bar-seq. This method has been previously validated in *S. cerevisiae* (Cook *et al.* 2008), yielding a high degree of overlap when compared to a strain-by-strain analyses (Jorgensen *et al.* 2002) (Figure 2A). In the current study, we screened a comprehensive set of 5470 heterozygous deletion diploid strains from the Merck DBC collection (Xu *et al.* 2007) for cell size defects. This collection covers 90% of the 6046 protein-coding open reading frames based on the current CGD annotation (Binkley *et al.* 2014). Two small cell size fractions were obtained by centrifugal elutriation and were used for these experiments (Figure 2B). Small cells and corresponding small deletion mutants are enriched in these fractions, while large cells strains are depleted. To determine mutant abundance in each fraction, genomic DNA of each pool was extracted and barcodes were PCR-amplified and sequenced. Abundance of each mutant in each fraction was appreciated by calculating the ratio of elutriated cells counts over counts of pre-elutriated cells.

To identify mutants with size defects, a two-step filter was applied. First, a size cut-off value was determined based on a benchmark set of conserved small (*sch9/SCH9*) and large (*cln3/CLN3 and cdc28/CDC28*) size mutants for which size was reduced or increased at least 12% as compared to the parental WT strain. Second, a normalized *Z*-score of 1.5 and −1.5 was used to identify both small (*whi*) and large (*lge*) size mutants, respectively. A total of 12 size mutants were excluded from our analysis, since they were found in both *whi* and *lge* datasets (Table S1). Microscopic examination revealed that these mutants had a remarkable size heterogeneity and grew predominantly as pseudohyphae. Based on these criteria, we identified 685 mutants that exhibited a size defect in both elutriated fractions. This includes 382 *whi* and 303 *lge* mutants (Table S2). As expected, *cln3/CLN3* and *cdc28/CDC28* mutants were identified as *lge* mutants, while *sch9/SCH9* was found among the smallest mutant in the elutriated pools. A total of 15 *whi* and 15 *lge* mutants were randomly selected and their size was measured by electrolyte displacement on a Coulter Z2 channelizer. The obtained data confirmed size defect in all 30 mutants examined (Table S3). Size phenotype of two heterozygous mutants, including the *rac1*/*RAC1* (Bassilana and Arkowitz 2006) and *sec15*/*SEC15* (Guo *et al.* 2016) previously shown as *lge* mutants, were confirmed by our analysis. However, the protein kinase C *pkc1*/*PKC1* mutant exhibited a *whi* phenotype in our investigation, while it was previously identified as large size (Paravicini *et al.* 1996). To clear up this contradiction, we have created new *pkc1*/*PKC1* mutants. At least five independent transformants were sized and the *whi* phenotype was confirmed for all of them (data not shown).

### Synthesis of ribosome and cell cycle are required for cell size homeostasis

GO enrichment analysis revealed that mutation in genes related to rRNA processing and ribosome biogenesis confer small cell size, while mutations of cell cycle genes result in *lge* phenotype (Figure 2, C and D and Table S2). Heterozygous deletion of genes of different functional categories related to protein translation, including rRNA processing (*CSL4*, *UTP7*, *DIS3*, *NOP53*, *FCF2*, *UTP23*, *DIP2*, *UTP15*, *SAS10*), ribosome exports (*RIX7*, *RRS1*, *NUP84*, *NUP42*, *RPS5*, *NOG1*), translation elongation (*RIA1*, *EFT2*, *CEF3*), and transcription of RNA Pol I and III promoters (*CDC73*, *RPB8*, *RPA49*, *RPB10*, *SPT5*, *RPA12*, *RPC25*), exhibited a *whi* phenotype. Heterozygous deletion mutation of structural components of both cytoplasmic (*RPL18*, *RPL20B*, *RPL21A*, *RPS5*, *UBI3*) and mitochondrial (*RSM24*, *RSM26*, *NAM9*, *MRPL20*) ribosomes decreased cell size. As in *S. cerevisiae*, mutants of the ribosome biogenesis regulator Sch9 and the transcription factor Sfp1 had a small size. Overall, as shown in other eukaryotic organisms, these data lead to the hypothesis that the rate of ribosome biogenesis or translation is a critical element underlying cell size control (Jorgensen *et al.* 2004). Haploinsufficient *whi* mutants also corresponded to catabolic processes associated mainly with ubiquitin-dependent proteolysis (*DOA1*, *GRR1*, *UBP1*, *UBP2*, *UFD2*, *SSH4*, *UBX7*, *RPN2*, *TIP120*, *TUL1*, *RPT2*, *PRE1*, *GID7*).

*Lge* mutants were predominantly defective in functions related to the mitotic cell cycle (Figure 2D and Table S2). These mutants include genes required for G1/S transition (G1 cyclin Cln3 and Ccn1, Cdc28 and Met30) suggesting that delay in G1 phase is the primary cause of their increased size. We also found that mutations in processes related to DNA replication (*ORC3*, *ORC4*, *MCM3*, *CDC54*, *RFC3*, *PIF1*, *SMC4*, *ELG1*), G2/M transitions (Hsl1, Cdc34) and cytoskeleton-dependent cytokinesis (*MYO5*, *INN1*, *SEC15*, *CDC5*, *CHS1*) conferred an increase of cell size. A similar observation was reported in *S. cerevisiae*, where a recent genome-wide microscopic quantitative size survey uncovered that mutants of the G2/M transition and mitotic exit fail to properly control their size. The large size of cell cycle mutants support the fact that cell growth and cell cycle are separate processes and cells continue to grow and increase their size without commitment to divide. Other investigations propose a model where, in addition to the G1-phase, size is sensed and controlled at G2/M checkpoint (Anastasia *et al.* 2012; King *et al.* 2013; Harvey and Kellogg 2003; Soifer and Barkai 2014). However, further analysis will be necessary to provide further insights into the presumptive linkage of each phase of the cell cycle and size homeostasis in *C. albicans*.

While a large proportion of *whi* mutants in *C. albicans* were related to ribosome biogenesis, inactivation of genes controlling translation initiation (*ASC1*, *SCD6*, *PAB1*, *GCD6*, *GCD2*, *SUI1*, *EIF4E*, *GCD11*) resulted in *lge* phenotype. A similar finding was reported in different genome-scale surveys of size phenome in *S. cerevisiae* (Jorgensen *et al.* 2002; Soifer and Barkai 2014). This large size phenotype in these mutants could be explained by the fact that regulators of Start onset, such as G1 cyclin Cln3 (Barbet *et al.* 1996; Polymenis and Schmidt 1997), are sensitive to the rate of translation initiation.

### Plasticity of size phenome and C. albicans fitness

Recent evidence has uncovered an extensive degree of rewiring of both *cis*-transcriptional regulatory circuits and signaling pathways across many cellular and metabolic processes between the two budding yeasts, *C. albicans* and *S. cerevisiae* (Lavoie *et al.* 2010; Li and Johnson 2010; Blankenship *et al.* 2010; Lavoie *et al.* 2009; Sellam and Whiteway 2016). In *S. cerevisiae*, a similar size haploinsufficiency screen was performed in heterozygous diploid strains of essential genes (Jorgensen *et al.* 2002). To assess the extent of conservation and plasticity of the size phenome, genes that were haploinsufficient for cell size in *C. albicans* were compared to their corresponding orthologs in *S. cerevisiae*. This analysis revealed a limited overlap between the two species with five *whi* (*rpl18a*, *sch9*, *rlp24*, *nop2*, *nog1*) and two *lge* (*rpt4*, *cln3*) mutants in common. In fact, genes with reciprocal size phenotypes were similar in frequency (the *whi* mutants *rpt2*/*RPT2* and *pkc1*\*PKC1* in *C. albicans* had *lge* phenotype in *S. cerevisiae*).

Interestingly, the corresponding homozygous deletion mutants of many *C. albicans* haploinsufficient size genes were shown to be required for virulence. A total of 69 size genes (representing ∼10%), including 47 small and 22 large size mutants, in our dataset were linked to *C. albicans* virulence or adaptation in the human host (Figure 2E). This suggests that cell size is an important virulence trait that can be targeted by antifungal therapy. Hypothetically, virulence defect in small size mutant could be linked to the reduced surface of the contact interface between *C. albicans*, with either host cells or medical devices in case of biofilm infections. Indeed, we have previously shown that the *whi* transcription factor mutant *ahr1* had attenuated virulence and exhibited a decreased attachment ability to abiotic surface such polystyrene, which consequently impaired biofilm formation (Askew *et al.* 2011). On the other hand, virulence defect in *lge* mutant could be associated with the fact that cells with large surfaces had a decreased lifespan which might impact their fitness and their viability inside the host (Yang *et al.* 2011; Mortimer and Johnston 1959).

While the link between *C. albicans* size and virulence remains uncharacterized, many investigations reported that many other fungal pathogens such as *Cryptococcus neoformans* and *Mucor circinelloides* adjust their cell size to access to specific niche in the host or to escape from immune cells (Wang and Lin 2012). In *C. albicans*, recent investigations have shown that large gastrointestinally induced transition cells, as compared to the standard yeast form, define the commensal form of this fungus (Pande *et al.* 2013). Furthermore, Tao *et al.* (2014) recently uncovered a novel intermediate phase between the White and *C. albicans* mating competent opaque phenotypes, called the Gray phenotype. The Gray cells are similar to opaque cells in general shape, however, they exhibit a small size and low mating efficiency. The Gray cell type has unique virulence characteristics, with a high ability to cause cutaneous infections and a reduced capacity in colonizing internal organs such as kidney, lung, and brain. Taken together, these lines of evidence emphasize the possible link between cell size and *C. albicans* fitness.

In summary, we provided the first comprehensive genome-wide survey of haploinsufficient cell size in a eukaryotic organism. In contrast to *S. cerevisiae*, where a similar screen was limited to essential genes (Jorgensen *et al.* 2002), our screen spanned the genome. A total of 300 (43.8%) dispensable genes and only 87 (12.7%) essential genes were haploinsufficient for size. Overall, our screen identified known conserved regulators (Sch9, Sfp1, Cln3) and biological processes (ribosome biogenesis and cell cycle control) required to maintain size homeostasis in this opportunistic yeast. We also identified novel *C. albicans* size-specific genes and provided a conceptual framework for future mechanistic studies. Interestingly, some of the size genes identified were required for fungal pathogenicity, suggesting that cell size homeostasis may be elemental to *C. albicans* fitness or virulence inside the host.

## Supplementary Material

Supplemental material is available online at www.g3journal.org/lookup/suppl/doi:10.1534/g3.116.037986/-/DC1.
