# Supplementary material for: Genome-Wide Screen for Haploinsufficient Cell Size Genes in the Opportunistic Yeast Candida albicans
Source: G3 (Bethesda). 2016 Dec 28;7(2):355–60. doi: 10.1534/g3.116.037986 (PMC5295585; doi:10.1534/g3.116.037986)
Supplement: Supplementary file 3 [file 355TableS2.docx]

Table S2. List of *whi* and *lge* haploinsufficient size mutants in *C. albicans* grouped according to GO biological process terms. (.xlsx, 81 KB)

[http://www.g3journal.org/lookup/suppl/doi:10.1534/g3.116.037986/-/DC1/TableS2.xlsx](http://www.g3journal.org/lookup/suppl/doi:10.1534/g3.116.037986/-/DC1/TableS1.xlsx)
