# Supplementary material for: Genome-Wide Screen for Haploinsufficient Cell Size Genes in the Opportunistic Yeast Candida albicans
Source: G3 (Bethesda). 2016 Dec 28;7(2):355–60. doi: 10.1534/g3.116.037986 (PMC5295585; doi:10.1534/g3.116.037986)
Supplement: Supplementary file 5 [file 355TableS3.docx]

Table S3. Confirmation of cell size defect in 15 *whi* and 15 *lge* mutants using the Z2-Coulter counter channelizer. (.xlsx, 34 KB)

<http://www.g3journal.org/lookup/suppl/doi:10.1534/g3.116.037986/-/DC1/TableS3.xlsx>
